# The effect of self-reflection on the outcomes of online clinical skills training: a comparative study

**DOI:** 10.1007/s10459-025-10425-8

**Published:** 2025-04-02

**Authors:** April Hoang, Stevie-Jae Hepburn, Alina Morawska, Matthew R. Sanders

**Affiliations:** 1 School of psychology, University of Queensland, Brisbane, Australia; 2 School of education, University of Queensland, Brisbane, Australia

**Keywords:** Self-reflection, Clinical skills, Online learning, Triple P, Professional training

## Abstract

Online learning is integral to pre- and post-licensure training across health and allied health disciplines. This randomized controlled study examined the impact of incorporating self-reflection prompts into an online clinical skills training module. A total of 88 health and social science students were randomly assigned to either a control group (*N* = 44) or an experimental group (*N* = 44). Both groups completed a brief, asynchronous online training module adapted from the Triple P - Positive Parenting Program, featuring a recorded lecture on clinical consultation skills. The experimental group received additional self-reflection prompts and completed written reflections, while the control group completed the training without reflective exercises. Participants in both groups completed pre- and post-training self-assessment surveys measuring knowledge, self-efficacy, and confidence, alongside a simulated client interview assessed using a standardized competency checklist for interpersonal and procedural skills. Results showed that participants in the experimental group demonstrated significantly greater improvements in knowledge acquisition, interpersonal skills, and procedural performance compared to the control group. These findings suggest that incorporating structured self-reflection activities in online training can enhance learning outcomes, promote deeper engagement, and improve self-awareness and clinical competence.

**Supplementary Information:**

The online version contains supplementary material available at 10.1007/s10459-025-10425-8.

## Introduction

Enhancing the quality of training in evidence-based intervention (EBI) is critical to ensure the effectiveness and sustainability of interventions addressing social, emotional, and behavioural issues at a societal level. High-quality EBI training equips professionals both pre and in-services with the necessary skills and knowledge to implement interventions accurately and effectively, thereby maximizing their impact on target populations (Dolcini et al., 2021). As the landscape of social and health care continues to evolve, professional development through high-quality training helps practitioners stay updated with the latest research and best practices, ensuring that they can respond effectively to emerging challenges (Beidas & Kendall, 2010). The health, allied health and educational professionals however, vary significantly in training background (e.g., psychology, early childhood education, social work, nursing) and operate in multiple settings, including health (primary care, mental health, and hospitals), educational settings (early childhood centres and schools), and social care/welfare sectors (Sanders et al., 2023). Training programs thus must be versatile and accessible, ensuring that individuals in multidisciplinary contexts can receive the necessary training. This approach not only accommodates the varied needs of the workforce but also ensures that EBIs can be effectively implemented across different settings to benefit individual social, emotional, and behavioural development (Sanders et al., 2023).

One solution to training that can help increase access to EBI training is online asynchronous training - a self-paced learning method where participants access pre-recorded content, modules, or resources independently, without the need for real-time interaction with instructors or peers. Although asynchronous training requires higher initial costs, it is more cost-effective for large-scale implementation and can reduce geographical barriers by allowing learners to complete training from their usual places of work or where permitted from home (Geiger et al., 2018). Asynchronous learning overcomes logistical barriers and encourages learners to be self-directed, engaging with various learning activities at times that suit their professional and personal commitments (Glogowska et al., 2011).

While the potential of online asynchronous training to expand the EBI workforce is clear, progress in incorporating asynchronous training into evidence-based training, particularly prior to COVID-19, has been slow. The COVID-19 pandemic acted as a catalyst for change and innovation (Stoehr et al., 2021), with online and blended learning no longer considered optional but essential for diverse professions. Consequently, many training courses quickly integrated online training activities into pre- and in-service training and professional development.

Research has consistently demonstrated the effectiveness of online training, showing that web-based training for EBI professionals can significantly improve participants’ skills (Jackson et al., 2018; Marano et al., 2020). When comparing online and traditional in-person training approaches, studies indicate that web-based, technology-assisted training can achieve comparable outcomes in terms of both knowledge acquisition and skill development (Soll et al., 2021). A comprehensive scoping review by Magill et al. (2022) further explored technology-based methods for training counselling skills, identifying several commercial companies and academic research centres focused on online training for behavioural health counselling. These programs were categorized based on their use of avatar or video client interfaces, and their level of interaction– ranging from fully interactive experiences to those utilizing pre-programmed, branch-logic interactions. The findings from this review highlighted the significant potential of technology-based training methods to effectively support the development of counselling skills, offering flexible and scalable options for training that can adapt to diverse learning needs.

While the initial findings on asynchronous training are promising, there is a clear need for further research to fully understand and enhance its quality and impact. Most existing studies primarily focus on assessing the overall effectiveness of online training, often overlooking the detailed processes and mechanisms that lead to these outcomes (Magill et al., 2022). Furthermore, there is a lack of specific insights into how different learning designs impact various aspects of learning outcomes in EBI in term of knowledge and skill acquisition. A more nuanced understanding of the effectiveness of different learning is crucial for optimizing training programs. This deeper investigation would allow educators and program developers to tailor asynchronous training strategies to maximize their impact, ensuring that they meet the diverse learning needs of participants and achieve the desired educational and practical outcomes.

Bennett-Levy (2006) introduced the declarative–procedural–reflective (DPR) model of skill acquisition, which provides a useful comprehensive framework for understanding the essential components of training for professionals. This model outlines three interconnected systems of knowledge processing that are crucial for developing expertise: declarative knowledge, procedural knowledge, and reflective knowledge. Declarative knowledge encompasses the understanding of facts and theoretical information, while procedural knowledge relates to the application of skills and techniques in real-world practice. Reflective knowledge, on the other hand, involves the capacity to analyses one’s experiences, integrate feedback, and adaptively refine one’s skills and approaches.

Bennett-Levy (2006) emphasizes that effective professional training relies on the integration of all three systems: declarative, procedural, and reflective. While declarative and procedural systems provide the foundational knowledge and practical skills, the reflective system is equally vital as it enables practitioners to critically evaluate their practice, continuously refine their methods, adapt to diverse clinical scenarios, and enhance therapeutic effectiveness (McLeod et al., 2020). Together, these systems create a comprehensive framework for developing professional competence.

Reflection is often activated in response to challenges or discrepancies between expected and actual outcomes in clinical practice, prompting the refinement of both declarative knowledge and procedural skills. This ongoing refinement leads to the development of increasingly sophisticated “when-then” rules, procedures, and competencies (Bennett-Levy & Finlay-Jones 2018).

To enhance reflection skills EBI, various training methods can be employed, including video demonstrations, facilitator feedback, peer feedback, and self-reflection activities (Mann et al., 2009). Among these, the self-reflection approach has gained increasing prominence especially in online training. This method empowers professionals to engage in continuous self-assessment and improvement, even without direct, real-time interaction, thereby supporting sustained professional development. It offers a scalable and flexible approach to fostering reflective practice without requiring substantial resources, while still upholding the rigor necessary for professional growth (Garrison, 2003; Sanders et al., 2023).

Self-reflection is built upon a self-regulatory framework that fosters self-awareness, self-sufficiency, self-efficacy, and continuous improvement (Bandura, 1991, 2014). It is highly impactful in professional training, contributing to both short-term and long-term outcomes (Ma et al., 2023; Turner et al., 2011). Bennett-Levy & Finlay-Jones (2018) suggest that self-experiential exercise is effective for enhancing the reflective system. A meta-synthesis by Gale and Schröder (2014) highlights the value of self-practice and self-reflection, demonstrating that these practices enhance the understanding of therapeutic models, communication skills, flexibility, creativity, self-awareness, and self-knowledge. Importantly, their findings suggest that self-reflection not only strengthens the reflective system but also contributes to the development of declarative and procedural knowledge, underscoring its multifaceted role in professional learning. However, Yan et al. (2023) indicates that while self-reflection exercises are beneficial, trainees often seek external feedback from peers, mentors, experts to enhance their learning. Trainees who actively engage in feedback-seeking behaviors may derive greater benefits from training programs compared to those who rely solely on course materials or personal reflections for feedback.

These contrasting perspectives raise critical questions about the standalone efficacy of self-reflection in promoting meaningful learning outcomes. While self-reflection can foster deep cognitive engagement, the absence of external feedback may limit its effectiveness in certain contexts. Given the growing demand for online asynchronous learning and the rapid expansion of training programs that incorporate self-reflection strategies, there remains a significant gap in understanding the true efficacy and mechanisms of self-reflection as a training method. Addressing this gap is essential for optimizing the design of EBI training programs to ensure they effectively meet the evolving needs of health, education and social care professionals.

Among the current EBI training program, Triple P– Positive Parenting Program is one of the few program that was accompanied with a comprehensive professional training model(Sanders & Murphy-Brennan, 2010; Sanders et al., 2003). Triple P training system was designed to equip students, early-career professionals, and experienced practitioners with the necessary skills to deliver parenting interventions effectively. Triple P promotes independent problem-solving and self-directed learning, incorporating personal goal setting, self-evaluation, and problem-solving as core components of its training model within a self-regulatory framework(Karoly, 1993; Mazzucchelli & Ralph, 2019). These principles align closely with Bennett-Levy’s (2006) self-reflective model, which underscores the role of self-reflection in developing professional competence.

In traditional Triple P training courses, which occur in-person or synchronously online, the self-reflective process is embedded within multiple teaching and learning activities, including role-playing activities, peer feedback, trainer feedback, and video demonstrations. Empirical evidence indicates that these interactive methods enhance practitioners’ knowledge, skills, and confidence in delivering interventions across both in-person and synchronous online formats (Sanders et al., 2023; Sethi et al., 2014).

However, similar to other skill-based training programs, transitioning to a fully asynchronous environment presents a key challenge- the absence of real-time feedback and supervision. Without immediate trainer debriefing or peer interaction, practitioners must rely solely on self-reflection to evaluate and refine their skills. This shift necessitates the evaluation of self-reflection as the primary strategy for facilitating effective reflective practice in asynchronous training, ensuring that learners still achieve meaningful skill acquisition and professional development.

### Current study

In this study, we aimed to investigate whether incorporating self-reflection techniques into a brief online asynchronous Triple P professional training module enhances learning outcomes compared to a control condition that does not include self-reflection activities. To address this aim, two research questions were proposed:

1. Does self-reflection improve trainees’ knowledge of the training content?
2. Does a self-reflection approach help improve trainees’ interpersonal and procedure skill.

For this study, a self-paced, single-module training protocol was utilized. The protocol was adapted from the training resources used in the Triple P professional training courses focusing on enhancing trainees’ skills in conducting an initial interview s part of an individually administered parent intervention (Primary Care Triple P (level 3) and Standard Triple P, level 4). The training aimed to equip practitioners with the ability to identify and understand child problem behaviours as reported by parents, enabling them to gather relevant contextual details regarding the nature, frequency, duration and intensity of the behaviour of concern. The training package included a pre-recorded video depicting social, emotional, and behavioural problems in parent-child interaction in a home setting, an explanation of the behaviour using a functional analysis assessment framework for identifying triggers (antecedents), the behaviours, and the factors that maintain or reinforce these behaviours (consequences), and a demonstration of a practitioner using this framework in a consultation with a parent or carer.

To assess the effectiveness of self-reflective activities, two versions of the training protocol were developed. The control condition included a standard training module with the pre-recorded video, theoretical explanation, and practitioner demonstration. The experimental condition featured an enhanced module in which the pre-recorded video was modified to incorporate self-reflective verbal prompts by the trainer/narrator throughout the training session (See Appendix 1). This experimental module included a structured self-reflection component divided into three parts. The first part involved retrospective reflection and skill gap identification, where participants engaged in self-assessment activities to identify knowledge gaps and areas for improvement. Specifically, they rated their confidence levels on a 10-point scale across key competencies, categorized the learnt skill areas as “easy” or “difficult,” and set specific goals for deliberate practice. The second part focused on self-evaluating their performance and goal setting, prompting participants to evaluate their pre-training performance, reflect on their areas requiring development, and establish actionable learning goals and objectives. These self-reflection activities are widely used in educational and clinical training contexts, with research demonstrating their effectiveness in fostering problem-solving, self-awareness, and professional learning (Watts, 2019). Such structured reflection mechanisms encourage practitioners to critically analyse their experiences, refine their clinical reasoning skills, and develop adaptive problem-solving strategies- essential elements of EBI training. Reflection-on-action, which involves reviewing one’s past performance, is also a key process that supports reflection-in-action, allowing practitioners to adjust their responses dynamically during client interactions (Bennett-Levy, 2006).

In addition to written self-reflection activities, the training module incorporated a five-minute guided mindfulness meditation focusing on diaphragmatic (abdominal) breathing (See Appendices 2 and 3). Mindfulness-based interventions are increasingly recognized as effective tools for enhancing self-awareness, regulating emotional responses, and improving cognitive focus (Bennett-Levy & Finlay-Jones, 2018). Diaphragmatic breathing activities, a key component of mindfulness training, have been shown to reduce physiological arousal and improve concentration (Gard et al., 2014). A systematic review by Leyland et al. (2019) found that mindfulness-based practices enhance self-regulation, particularly through attentional control and emotion regulation. Additionally, research suggests that mindfulness supports cognitive flexibility, enhances empathy, and improves appraisal of emotional experiences (Farrar et al., 2022; Hölzel et al., 2011).

By integrating self-reflective prompts and mindfulness-based activities, this training module aimed to simulate key aspects of reflective learning in an asynchronous format. We hypothesized that participants who engaged with self-reflection activities, would show greater improvements in post-training assessments and simulated skill tasks compared to those in the control group, who do not receive such prompts. This study aimed to provide insights into optimizing asynchronous training methods by understanding the role of self-reflection in enhancing professional competencies.

## Method

### Design

This study employed a 2 (training condition: self-reflection prompt vs. no self-reflection prompt) × 2 (time: pre and post) randomized controlled trial design. Block randomization was used to ensure equal allocation of participants while allowing data collection to occur promptly, reducing the risk of participant dropout, and facilitating the attainment of the desired sample size. Once 20 participants were recruited, they were randomly assigned to either the intervention or control condition and immediately received training.

### Power analysis

A priori power analysis was conducted using G*Power to determine the required sample size for a 2 (training condition: self-reflection prompt vs. no self-reflection prompt) × 2 (time: pre and post) randomized controlled trial design. Assuming a small effect size (*d* = 0.25) to be found at an α level of 0.05, and 80% power, the required sample size was estimated to be approximately 34 participants per group (total *N* = 68). With 88 participants, the study was adequately powered to detect meaningful differences in training outcomes while accounting for potential dropout or variability in effects.

### Procedure

The study received ethical approval from the University (blinded for review) Human Research Ethics Committee (Approval number blinded for review). Health, behavioral and social sciences students were recruited via university social media platforms, online coursework noticeboards, email distribution by course coordinators and the University (blinded for review) Research Participation Scheme. Data collection took place between February and August 2023. The participants registered their interest in the project via email and the project team provided the participant information sheet prior to participation. The Triple P training system has been shown to effectively enhance trainees’ skills and confidence in delivering the program, regardless of their educational background, level of education, or primary language (Sanders et al., 2023). Thus, to be eligible for the study, participants were required to be enrolled in an undergraduate or postgraduate health or social science degree. No other inclusion criteria were applied to the participant group.

To ensure that all participants received the same exposure duration and learning opportunities- without variations introduced by differences in self-paced engagement, external assistance, or extended review time- a structured experimental session was conducted. A research assistant facilitated the session, acting as a timekeeper to standardize the duration of the learning experience across participants. Prior to the session, participants received an email with key session details, including the time, date, participant ID, and Zoom link. At the start of the session, they completed a pre-assessment survey via a Qualtrics link, followed by an observational skill assessment. This assessment required participants to conduct an intake interview with a simulated parent, played by the research assistant, who expressed concerns about their child’s behaviour. The session was recorded via Zoom to ensure consistency in data collection, and all participants were presented with the same case scenario.After the pre-training assessment, participants engaged in the training session, facilitated by the research assistant. Upon completion, they underwent a post-training assessment, which was identical to the pre-training assessment. This included a post-training survey with the same measures as the pre-assessment and a second skill observation case study, conducted with the same research assistant to maintain consistency in skill evaluation. The entire experimental session lasted approximately 70 min. To acknowledge their participation, each participant received a $30 AUD electronic gift card. The structured workflow of the experimental session is outlined in Table 1.

**Table 1:** Experimental procedure

#	Control group	Experimental group	Duration
1.	Pre-training surveys Demographics, Theoretical Knowledge quiz, The Practitioner Consultation Skills Checklist		5 min
2.	Pre-training skill observation Role-play intake interview with the research assistant		5–10 min
3.	Training protocol Theoretical material– pre-recorded lecture (no prompts), and written materials	Training protocol Theoretical material– pre- recorded lecture with self-reflective prompts, and written materials	18 min
	Reading and reviewing materials	Worksheet - written reflection activities Mindfulness activity	10 min
5	Post training surveys Demographics, Theoretical Knowledge quiz, The Practitioner Consultation Skills Checklist		5 min
6	Post-training skill observation Role-play intake interview with the research assistance		5–10 min

### Measures

Pre- and post-training surveys were administered to ascertain changes in knowledge, and participants’ self-reported of their confidence in applying the learnt skills. Observational data of recorded role-play exercises were also included to determine the impact of self-reflection on participants’ actual skill development and performance. Written reflection activities completed by participants in the experimental group were also included.

### Self-report measures

#### Demographics

Demographic questions covered participants’ age, gender, university program or degree, health or social science discipline, and any prior training in evidence-based parenting.

#### Theoretical knowledge quiz

A quiz with four single-choice questions was used to measure participants’ knowledge of evidence-based parenting. Sample of questions include: “*Why is it important to identify what is happening when the problem behaviour begin/stop*?” or “*What is the purpose of the framework for discussing problem behaviour*”? of which the participants were given four options to choose from. These questions were designed to test knowledge of the key principles and concepts of intake interview and has been used regularly in Triple P program as part of the accreditation quiz.

#### The practitioner consultation skills checklist

The Practitioner Consultation Skills Checklist (PCSC) (Turner & Sanders, 1996) is a tool used to assess participants’ confidence and self-efficacy in consultations with parents or carers, particularly concerning behavioural issues and the implementation of parenting strategies. It includes two subscales: confidence in conducting consultations and self-rated proficiency in applying learned skills. The confidence subscale measures participants’ perceived ability to engage in consultations, with items such as *“How confident are you in conducting parenting consultations about child behaviour?”* The proficiency subscale on the other hand asked participants’ self-evaluation of their ability to apply specific consultation skills such as *“Clarifying the specific problem*,* its frequency*,* intensity*,* antecedents*,* and consequences”* or *“Discussing causes of children’s behavioural problems.”* Responses are rated on a 7-point Likert scale, with higher scores reflecting greater confidence and proficiency. The PCSC has demonstrated strong psychometric properties, supporting its reliability and validity (Sanders et al., 2023; Sethi et al., 2014). In this study, six items specifically assessing confidence and self-evaluation of interview skills were used. The version of the PCSC used exhibited high internal consistency, with a Cronbach’s alpha of 0.95, indicating excellent reliability.

### Observational data

Observational data were used to assess improvements in consultation skills between pre- and post-training role-play activities with a simulated parent/client. A competency checklist (10 items), adapted from the Participant Skills Checklist (Turner, Sanders, & Markie-Dadds), evaluated both interpersonal (2 items) (e.g., communication, empathy, active listening) and procedural skills (8 items) (e.g., use of open-ended and probing questions). Each of the eight procedural items was rated on a scale from 0 (not demonstrated) to 10 (highly proficient), with proficiency defined by detailed skill descriptors (See Appendix 4). The checklist was refined through iterative discussions among three research team members with expertise in consultation skills. Total scores for interpersonal and procedural skills were calculated, along with an overall Interview Competency Score by summing both components.

Two research assistants were trained to score the observational data against the pre-determined criteria. Each research assistant trialled the criteria and met to discuss any discrepancies in consultation with one of the research team. After trialling the criteria and calibrating the scoring process, each research assistant independently viewed each recording and scored the skill demonstrated by each participant in the pre- and post-training simulated interview. Interrater reliability was assessed using the Intraclass Correlation Coefficient (ICC) and Pearson correlation. The ICC indicated excellent reliability between raters, with a value of 0.89. Pearson correlation analysis also demonstrated a strong, statistically significant correlation between the two raters’ scores (*r* =.80, *p* <.001).

## Results

### Participants

A total of 132 participants registered for the study and received study information. Of these, 109 completed the baseline survey. However, some participants either failed to attend the sessions or cancelled prior to the scheduled time (*N* = 21), resulting in a final sample of 88 participants (Control: *N* = 44; Experimental: *N* = 44).

Table 2 provides a summary of the participants’ demographics, including age, current year of enrolment, and field of study. Most participants were aged 20–24 years (*N* = 56, 63.64%), female (*N* = 76, 86.36%), and undergraduate students (*N* = 64, 72.72%). Among the undergraduates, a significant proportion were majoring in Psychology (*N* = 27, 30.68%) and Education (*N* = 17, 19.31%). Among them, 15 participants (17.04%) failed to disclose their Field of study.

**Table 2:** Demographics of participant group (*N* = 88)

Characteristic	*Control* (*N =* 44)		Experiment (*N =* 44)	
	*N*	%	*N*	%
Age (years)				
Under 20	0	0.00	1	2.30
20–24	31	70.50	25	56.80
25–29	9	20.50	6	13.60
Over 30	4	9.09	12	27.20
**Gender**				
Male/man	15	34.10	3	6.80
Female/woman	28	63.60	39	88.60
Non-binary/third gender	1	2.30	2	4.50
**Current enrolment**				
Third year of college	1	2.30	1	2.30
Undergraduate degree– 2nd year	11	25.00	9	20.50
Undergraduate degree– 3rd year	18	40.90	12	27.30
Undergraduate degree– 4th year	3	6.80	9	20.50
Master’s program– 1st year	7	15.90	6	13.60
Master’s program– 2nd year	2	4.50	6	13.60
Doctorate/PhD/MD programs	2	4.50	1	2.30
**Field of study**				
Audiologist	1	2.27	0	0.00
Chiropractor	1	2.27	0	0.00
Dietitian	0	0.00	2	4.55
Exercise physiologist / Sport Science	0	0.00	1	2.27
Occupational Therapist	2	4.55	4	9.09
Pharmacists	0	0.00	1	2.27
Physiotherapists	0	0.00	1	2.27
Psychologist	12	27.27	15	34.09
Public Health	5	11.36	1	2.27
Registered Nurse	3	6.82	1	2.27
Social Worker	2	4.55	2	4.55
Speech Pathologist	1	2.27	1	2.27
Education	11	25.00	6	13.64
Profession not specified	6	13.64	9	20.45

### Data analysis

An inspection of participants demographics revealed a higher number of male participants in the experimental group compared to the control group (*N* = 15 vs. *N* = 3; *X²* = 10.14, *p* <.05). As previous studies have indicated the potential influence of gender on training outcomes (Sanders et al., 2023), an analysis of gender effects on baseline scores across each group was conducted. Results indicated that there was a significant difference between males and females on self-efficacy (*F*(1) = 11.96, *p* <.05); confidence (*F*(1) = 16.32, *p* <.05) and total observational scores (*F*(1) = 5.52, *p* <.05) at baseline. We thus conducted a thorough comparison of baseline scores which indicated that the experimental group scored significantly higher than the control group not in self-reported efficacy but in observational skills at pre-training (see Table 3). A series of ANCOVAs were thus applied to evaluate post-training effects. In the primary ANCOVA models, we controlled for baseline scores of the respective outcome variables to account for pre-existing differences, ensuring that post-training effects were attributed to the intervention rather than initial skill levels. Gender was not included as a covariate, as its influence was already reflected in baseline scores, which were statistically controlled in the ANCOVA models. This approach prevents overadjustment while ensuring that differences in post-training outcomes are attributable to the intervention rather than pre-existing disparities. Effect sizes were reported using Cohen’s d, where values of *d* ≤ 0.20 indicate a small effect, 0.21 to 0.79 represent a medium effect, and *d* ≥ 0.80 reflect a large effect (Cohen, 1988). This classification provides a standardized way to interpret the magnitude of observed differences, offering insights into the practical significance of the findings beyond statistical significance.

**Table 3:** Intervention outcomes for the control and experimental groups

	Experiment (*N* = 44)		Control (*N* = 44)			Time 2 Condition Effect F(df), *p*	Effect size
	Time 1 M (SD)	Time 2 M (SD)	Time 1 M (SD)	Time 2 M (SD)	Baseline difference T(df), *p*		
**Self-report data**							
Knowledge	1.61 (0.81)	2.39 (0.65)	1.86 (0.77)	1.93 (0.82)	*t(86)=-1.48, p >.05*	*F*(1,86) = 6.98, *p* <.05*	0.59 [0.07, 1.11]
Confidence	6.27 (3.08)	9.41 (2.07)	5.14 (2.46)	9.50 (2.32)	*t(86) = 1.91, p >.05*	*F*(1,86) 1.908, *p* >.05	0.46 [-0.05, 0.97]
Self-efficacy	13.66 (5.96)	18.02 (4.12)	11.82 (5.59)	19.45 (4.42)	*t(86) = 1.49, p >.5*	*F*(1,86) 0.79, *p* >.05	0.00 [-0.51, 0.51]
**Observation data**							
*Interpersonal skills - total*	8.92 (4.70)	12.43 (4.57)	6.31 (4.19)	9.03 (4.50)	*t(86) = 2.75, p <.5*	F(1,86) = 4.33; *p* <.05*	0.46 [-0.05, 0.97]
Confidence to deliver	4.47 (2.09)	6.59 (2.28)	3.23 (1.92)	4.62 (2.29)	*t(86)* = 2.89, *p* <.05	*F*(1,86) = 7.22, *p* <.05*	0.59 [0.07, 1.11]
Empathy and active listening	4.45 (2.97)	5.84 (2.66)	3.08 (2.53)	4.41 (2.52)	*t(86) = 2.34*, *p* <.05	*F*(1,86) = 1.45, *p* >.05	0.29 [-0.22, 0.80]
*Procedure skills- Total*	23.27 (10.05)	37.50 (10.70)	17.42 (8.70)	29.85 (10.57)	*t(86) = 2.92, p <.05*	*F*(1,86) = 3.886, *p* =.052	0.41 [-0.10, 0.92]
Using open-ended questions to clarify the target problem	5.98 (2.60)	7.41 (2.19)	5.11 (3.43)	6.95 (3.39)	*t(86) = 1.33, p >.05*	*F*(1,86) = 0.001, *p* >.05	0.00 [-0.51, 0.51]
Helped the parent come up with a specific description of the problem behaviour	3.42 (2.36)	5.80 (2.22)	2.67 (2.14)	4.33 (2.33)	*t(86) = 1.56*, *p* >.05	*F*(1,86) = 6.81, *p* <.05*	0.55 [0.03, 1.07]
Exemplification probe question to ask parent to share a specific example	2.27 (2.46)	4.49 (2.10)	1.06 (1.97)	4.05 (2.55)	*t(86) = 2.56*, *p* <.05	*F*(1,86) = 0.059, *p* >.05	0.05 [-0.46, 0.56]
Used more specific questioning techniques to identify the consequences of the behaviour	0.83 (1.24)	4.06 (2.46)	0.86 (1.48)	2.47 (2.21)	*t(86)=-0.12*, *p* >.05	*F*(1,86) = 10.162, *p* <.05*	0.70 [0.18, 1.22]
Used more specific questioning to identify the antecedents of the behaviour	4.64 (2.61)	3.52 (2.71)	3.67 (2.71)	3.07 (2.56)	*t(86) = 1.70*, *p* >.05	*F*(1,86) = 0.140, *p* >.05	0.00 [-0.51, 0.51]
Used more specific questioning to obtain any additional useful information	3.51 (2.03)	3.48 (2.59)	2.33 (2.09)	2.60 (2.29)	*t(86) = 2.69*, *p* <.05	*F*(1,86) = 1.299, *p* >.05	0.29 [-0.22, 0.80]
Summarised the incident and checked the parent’s reaction	0.58 (1.70)	4.56 (2.80)	0.07 (0.45)	2.93 (3.51)	*t(86) = 1.93*, *p* >.05	*F*(1,86) = 3.934, *p* =.056	0.41 [-0.10, 0.92]
Checked for generality	2.05 (2.48)	4.19 (2.67)	1.65 (2.40)	3.45 (2.74)	*t(86) = 0.76*, *p* >.05	*F*(1,86) = 1.145, *p* >.05	0.20 [-0.31, 0.71]
**Total Interview technique**	32.19 (14.00)	49.93 (14.29)	23.73 (11.85)	38.89 (13.98)	*t*(86) = 3.06, *p* <.05	*F*(1,86) = 4.12, *p* = < 0.05	0.46 [0.00, 1.00]

### Missing data analysis

Among the main variables, only four contained missing data, with a total of four missing values (0.22%). Missingness below 5% is considered non-significant (Schafer & Graham, 2002); therefore, no imputation or data correction was performed. The missing values were excluded from the relevant analyses using listwise deletion. This method has been found to perform better than mean replacement in tests such as ANOVA and ANCOVA, as it preserves the natural variability of the data and reduces the risk of bias (Cheema, 2014).

### Main findings

#### Pre- and post-training self-report data

Following training, significant differences were shown for the Theoretical Knowledge Quiz, with the experimental group performing better than the control group, *F*(1,86) = 6.98, *p* <.05, with a medium effect size *Cohen’s d* = 0.59. No significant differences between the two groups on the self-reported measures of confidence and self-efficacy were found (See Table 2).

#### Pre- and post-training observational data

Observational data indicated that, overall, the experimental group showed better consultation skills than the control group post training. The total Interview Competency score of the experimental group post-training was significantly higher than that of the Control group, after controlling for pre-training scores (*F*(1,86) = 4.12, *p* <.05), with a medium effect size (*Cohen’s d* = 0.46). Further breaking the scores down, it was observed that the experimental group demonstrated significantly higher Interpersonal skills than the control group post-training; especially in their confidence in delivering consultations which was significantly greater than that of the control group (*F*(1,86) = 7.22, *p* <.05) with the effect size of *Cohen’s d* = 0.59 (see Table 2).

While the improvement in total Procedural skills did not reach statistical significance between the two groups, significant differences were observed in two specific procedural skills: “*Helping the parent come up with a specific description of the problem behaviou*r” and “*Using more specific questioning techniques to identify the consequences of the behaviour*.” The experimental group showed significantly higher scores on these skills post-training compared to the control group. The effect sizes of changes were medium for both skills with *Cohen’s d* = 0.55 and 0.70 respectively.

Additionally, the items “*Summarizing the incident*” and the overall interview techniques score approached statistical significance, suggesting a positive trend towards improvement in these areas for the experimental group (see Table 2).

## Discussion

This study aimed to investigate whether incorporating self-reflection techniques in a brief online asynchronous Triple P professional training module would enhance learning outcomes compared to a control condition that did not include self-reflection activities. The study findings provide partial support for the hypothesis. While no significant differences were observed between the experimental and control groups concerning their self-reported measures of confidence and self-efficacy, significant differences emerged in terms of their knowledge of evidence-based parenting and observation of their actual skills. These significant findings regarding participants’ knowledge are consistent with existing literature, suggesting that self-reflection can be beneficial for enhancing both conceptual or theoretical knowledge and procedural or technical skills (Gale & Schröder, 2014).

An important finding of this study, which deserves further discussion, is that compared to the control group, participants in the experimental group demonstrated a marked improvement in their confidence to interview parents and showed significant improvement in certain procedural skills, as evidenced by the observational data. These improvements were most pronounced in tasks such as assisting parents in articulating a specific description of problematic behaviours and employing more targeted questioning techniques to identify the consequences of such behaviours.

The improvement of participants’ ability of the experimental group relative to control group to help parents accurately identify and understand their children’s behavioural issues and the corresponding consequences as demonstrated via the observational data is an important finding. These tasks are critical in the initial interview because they enable practitioners to guide parents in recognizing and articulating their children’s behaviours more clearly (Head & Abbeduto, 2007); Sanders & Mazzucchelli, 2017). This clarity is essential for understanding the current problem and for developing an effective parenting approach to manage the problem. Effective questioning in these areas thus may have reduced the need for follow-up inquiries, thereby streamlining the consultation process. For instance, practitioners who can successfully aid parents in providing a detailed and specific description of a child’s behaviour are less likely to need to ask for additional examples, thus saving time and focusing the consultation. Similarly, when practitioners are proficient at exploring the consequences of a child’s behaviour, the need for extensive questioning about the context of the behaviour might have diminished This ability to narrow down the focus and efficiently address the issues at hand not only enhances the quality of the consultation but potentially can be crucial in helping parents become more active participants in the behaviour management process (Schumacher & Madson, 2014), ultimately leading to better long-term outcomes for the child and family through developing more highly targeted and effective parenting responses.

Although the mechanism behind our findings, especially the fact that changes in observational skills are evident requires further exploration, it is possible that reflective practices may enable participants to thoroughly consider and assess their experiences, allowing them to identify key areas for improvement. This explanation is aligned with the reflective practices literature that suggests individuals may be better equipped to critically analyse their performance, recognize gaps in their knowledge or abilities, and implement necessary changes to enhance their professional growth and competence (Mann et al., 2009). This process thus not only builds confidence but also ensures that learning is translated into actionable skills, which is critical for the sustainability and effectiveness of training programs over time (Travers et al., 2015). Importantly, these significant differences in consultation skills between the experimental and control groups were observed despite the short (27 min), non-intensive nature of the training module, which covered relatively straightforward knowledge and skills. These observations suggest that self-reflection activities can significantly improve training outcomes, even in a relatively brief timeframe.

While our observational data support the view that participants improved their skills, this significant difference was not reflected in self-reported data. It is important to note that while the observational data measured participants’ skills, the self-reported data captured their confidence levels. The disparity between these findings may suggest that participants are improving in their actual skills, but their confidence has yet to catch up. This gap is not unusual, as Hitzeman et al. (2020) highlighted that learners often underestimate their capabilities, leading to less accurate self-assessments which could be due to various biases, such as social desirability, overestimation, or underestimation of one’s abilities (Brenner & DeLamater, 2014; Poulton, 1977). These biases can obscure the true impact of training on self-confidence, which may require more practice and extended implementation to fully develop and align with actual skill gains. This underscores the importance of a more nuanced understanding of the progression of skill development and confidence building after training, as self-efficacy is also a key driver of sustained application of learned skills (Turner et al., 2011). While participants’ skills may have improved quickly after training, their confidence might be more complex and take longer to become evident,

## Implications and future directions

Our study offers several key implications for the design and delivery of EBI training. First, it underscores the benefit of incorporating self-reflection activities into EBI training programs as a structured method for individuals to enhance their practice. Self-reflection allows trainees to critically assess their own skills, identify areas for improvement, and track their progress over time. By engaging in self-reflection, individuals can develop a deeper understanding of the intervention techniques and their practical application, which can lead to more effective and adaptive use of these techniques in real-world settings (Cushway, 2009; Hughes, 2013).

This approach can be particularly effective even within very brief interactions, as demonstrated by our findings. By integrating structured self-reflection into EBI training, programs can ensure that trainees are consistently thinking critically about their practice, take ownership of their professional development, actively seek feedback, and remain open to new ideas and approaches. This mindset is crucial in the rapidly evolving field of evidence-based practice, where ongoing adaptation and responsiveness to new research findings are essential for maintaining high standards of care (Lim & Low, 2008). However, future research should seek to clarify which specific activities used in training to determine which methods are best suited to deliver different domains of training, such as knowledge acquisition, procedural skills, or interpersonal skills. There is also considerable interest in combining self-reflective activities with other forms of reflection, such as video reviews, to significantly improve professionals’ abilities to manage consultations effectively.

A second important implication of our study concerns the types of data used to evaluate the effectiveness of EBI training. The discrepancies observed between self-reported and observational data highlight the distinct information provided by different measures and suggest potential differences in the rate of improvement between participants’ actual skills and their confidence levels. This underscores the need for more nuanced and long-term data collection methods in future studies to better account for such discrepancies. A mixed-method approach, integrating both self-reports and objective performance measures over time, would provide a more comprehensive and accurate understanding of how training influences both skill acquisition and self-efficacy. This approach would allow trainers to track improvements in both areas and address any lag between skill development and confidence. Moreover, leveraging technology-enhanced methods, such as video analysis or digital tracking of performance, can further enrich the data collection process, offering clearer insights into participant progress and helping to align subjective self-perceptions with objective performance indicators (Jenny et al., 2021). By employing these methods, future research can more effectively capture the full scope of training outcomes and ensure a more precise evaluation of EBI training programs.

By bridging the gap between self-reported perceptions and observed outcomes, these advanced data collection methods can significantly enhance the understanding of how training translates into actual skill performance. They allow for more accurate assessment of training efficacy, leading to insights that can inform the continuous refinement of training programs. From an educational design perspective, the findings indicate that participants needed more scaffolding or support to manage time, use probing questions and checking for generality, thus in future iterations of the program this content or these skills can be emphasised and, self-evaluation may be used to inform instructional strategies.

## Strengths and limitations

A key strength of this study is its innovative approach, as it incorporates both self-report and observational data to assess the impact of self-reflection in training programs. This is among a small number of studies to do so. The inclusion of observational data allows for a more comprehensive and objective evaluation of training outcomes, capturing aspects of participant behaviour that self-reports alone might miss. By combining self-report measures with observational data, this study provides a richer, more nuanced understanding of how self-reflection influences both perceived and actual performance. This methodological approach enhances the validity of the findings and provides a more robust framework for evaluating the effectiveness of training interventions. Also, it is worth noting that Triple P is a highly effective program, which has been demonstrated to be effective in enhancing practitioners’ confidence and efficacy in delivering intervention regardless of their background and demographics (Sanders et al., 2023). The fact that we found significant differences between two groups with such a small modification to the program indicates high potential of the self-reflection component in enhancing training outcomes. Future studies, could further provide a clearer understanding of how impactful self-reflection activities may be in enhancing training outcomes.

Despite its strengths, this study has several limitations that warrant consideration. A key limitation is that the outcome measures were based on adapted instruments that have not yet undergone formal validation.Although the brief Theoretical Knowledge Quiz, Practitioner Consultation Skills Checklist, and skill observation checklist were developed from widely used tools within the Triple P training process, the specific versions used in this study have not been thoroughly evaluated across diverse samples and contexts. Consequently, the findings should be interpreted with caution, as the measures’ reliability and validity may vary in different settings. Future research should prioritize the validation of these measures to strengthen their reliability, generalizability, and applicability in various training settings.

A limitation of this study is the baseline differences observed between groups, particularly in terms of gender, despite our efforts to carefully randomize participants. Such differences are not uncommon in randomized studies and can arise from sample size variations or random chance (De Boer et al., 2015). To mitigate the impact of these disparities, we employed statistical methods to control for potential confounding variables.

Larger sample sizes in future studies may allow for a more in-depth exploration of gender differences in training outcomes. Educational and learning literature suggests that males and females differ in self-efficacy and learning strategies (Huang, 2013; Sanders et al., 2023), potentially leading to varied responses to interventions. While we did not have sufficient statistical power to investigate these gender-specific effects in the present study, future research should aim to address this gap to deepen our understanding of how gender influences training outcomes both in term of skills and their confidence.

Furthermore, the generalizability of this study may be limited by the specific population we recruited, which primarily consisted of students. This decision was based on prior findings indicating that in Triple P training can accommodate a multidisciplinary workforce with diverse educational backgrounds and training experiences with minimal training differences among groups (Sanders et al., 2023; Sethi et al., 2014). However, this pattern has been observed primarily in in-person training contexts. In the case of asynchronous online training, different dynamics may be at play. University students, who are more actively engaged in academic learning and familiar with online education platforms, may adapt more readily to this training format. In contrast, in-service professionals, who may not engage in regular structured learning, could face greater challenges in navigating the online training environment. Future research should explore whether training outcomes differ between pre-service and in-service practitioners in asynchronous learning contexts.

Additionally, the relatively short duration of the intervention may constrain the ability to capture the long-term impact of self-reflection on skill development and professional practice. Future research could enhance understanding by incorporating extended follow-up periods and engaging more diverse participant samples to evaluate the sustainability and broader applicability of training outcomes. Given that skill acquisition and refinement require continuous practice, ongoing implementation support and opportunities for applied learning should be integrated into professional development frameworks to reinforce and sustain training effects.

## Conclusion

The inclusion of self-reflection in training programs can markedly improve technical skills and knowledge among learners. Although it may not directly enhance confidence its impact on procedural skills and reflective practice is substantial. This study underscores the value of self-reflection in professional training, suggesting that even brief, non-intensive modules can benefit from its inclusion. Future research should continue to explore the specific mechanisms through which self-reflection enhances training outcomes and how these can be optimized across various training contexts.

## Electronic supplementary material

Below is the link to the electronic supplementary material.

Supplementary Material 1

## Data Availability

The data that support the findings of this study are available from the Parenting and Family Support Centre, UQ, but restrictions apply to the availability of these data for confidentiality of participant information. The data are, however, available from the authors upon reasonable request.
