# Supplementary material for: The effect of self-reflection on the outcomes of online clinical skills training: a comparative study
Source: Adv Health Sci Educ Theory Pract. 2025 Apr 2;30(5):1621–39. doi: 10.1007/s10459-025-10425-8 (PMC12572028; doi:10.1007/s10459-025-10425-8)
Supplement: Supplementary file 1 — Supplementary Material 1 [file 10459_2025_10425_MOESM1_ESM.docx]

Appendix 1: Screenshot of the self-reflection exercise guidance


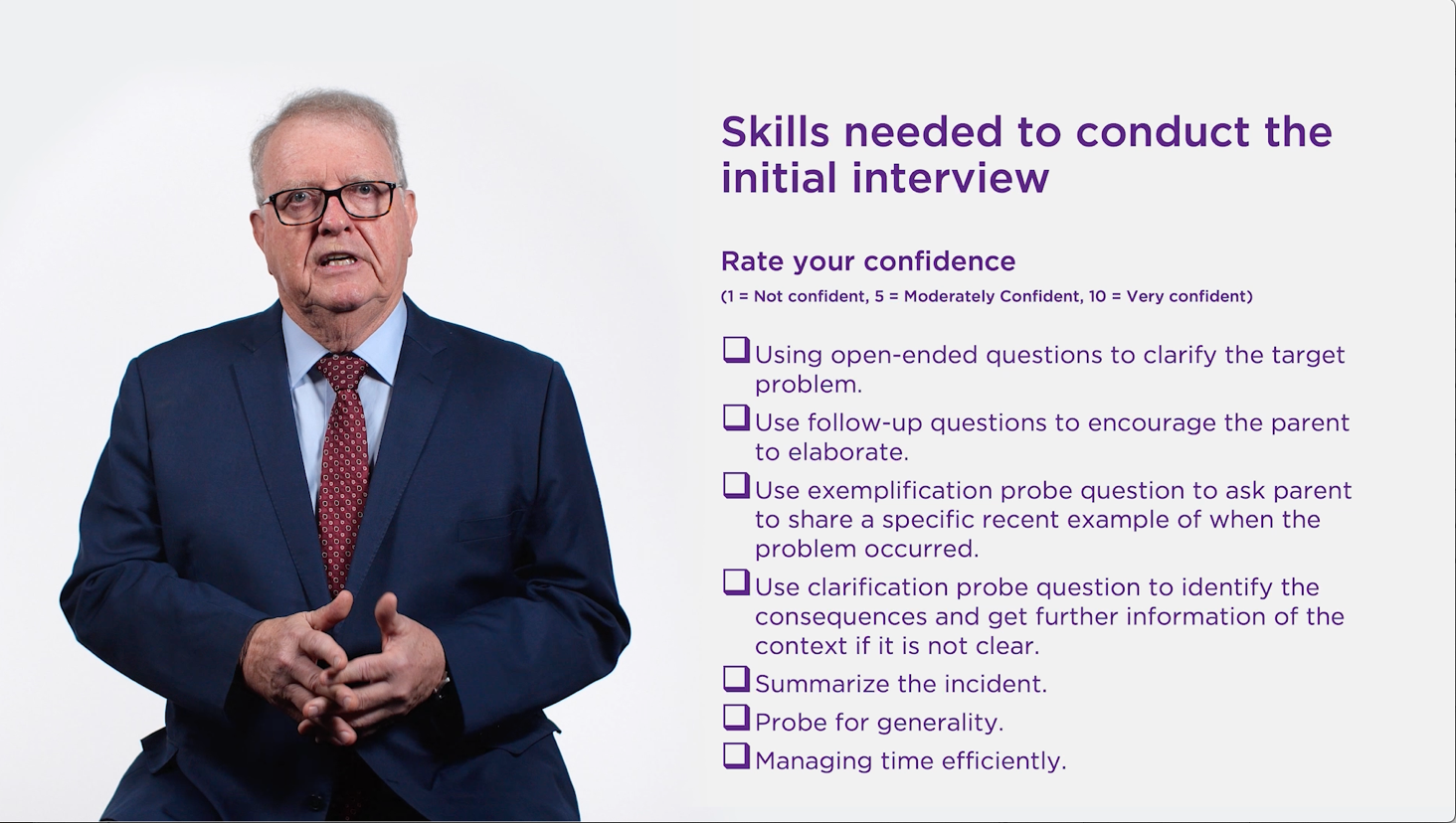


Appendix 2. Screenshot of the Mindfulness breathing guide


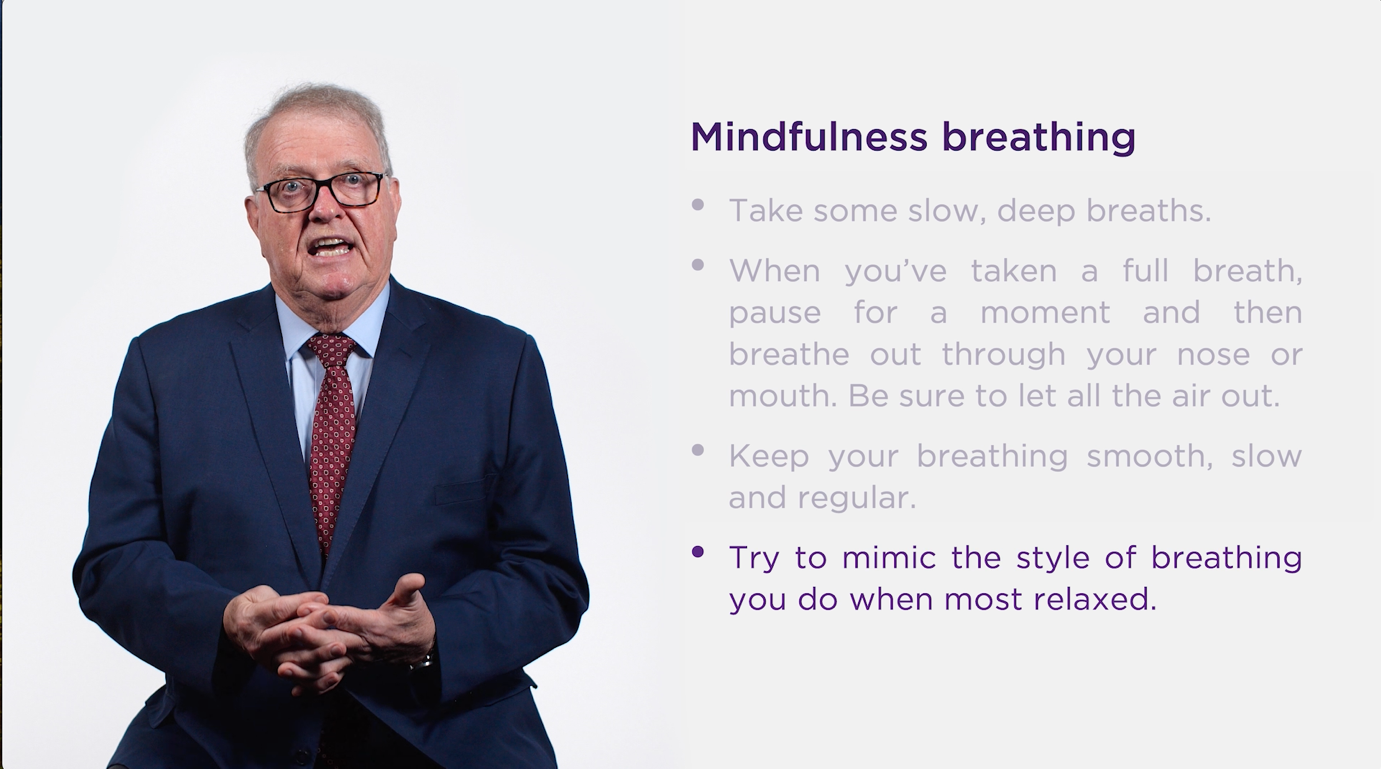


**Appendix 3: Screenshot of the Mindfulness Breathing Exercise in Sequences**

This appendix presents a series of screenshots illustrating the mindfulness breathing exercise as presented to participants during the intervention. The sequences demonstrate the step-by-step process, including instructions, breathing cues, and visual or auditory prompts designed to guide participants through the exercise.


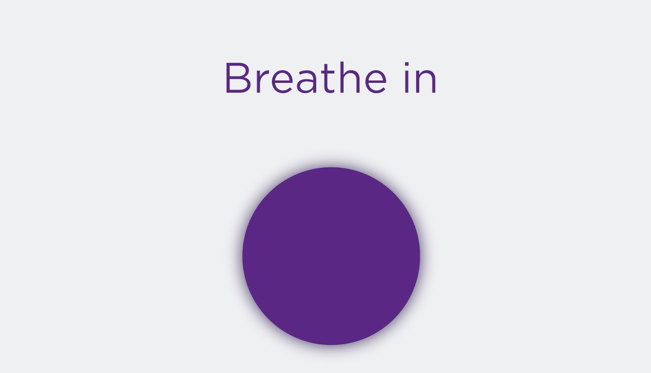

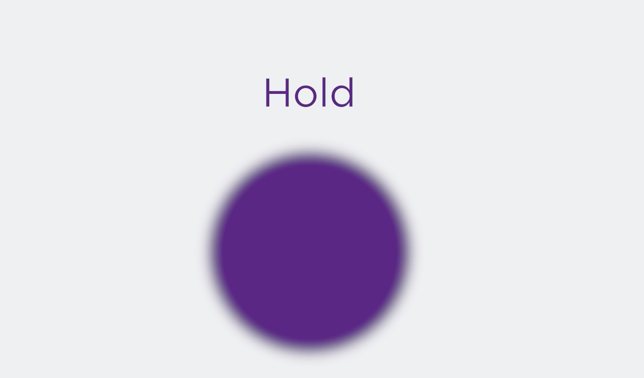

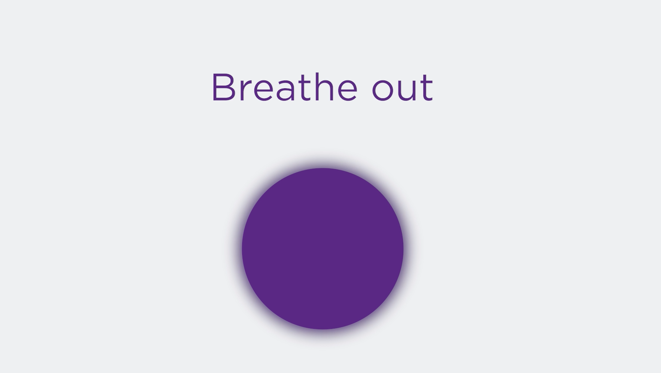


Appendix 4. Observational rating checklist

| **Interpersonal Skill** | | Description | | | | | | | | | | | | | | | | | |
| --- | --- | --- | --- | --- | --- | --- | --- | --- | --- | --- | --- | --- | --- | --- | --- | --- | --- | --- | --- |
| A | Confidence to Delivery | During the interview, a proficient practitioner demonstrated a sophisticated ability to communicate and relate to the parent. The practitioner:   1. commenced the interview with a professional, confident greeting and introduction to the parent/carer. 2. demonstrated their ability to engage with the parent in a professional, conversational manner and referred to the child’s name when speaking with the parent. 3. adjusted their tone and language choice appropriately when responding to the parent. 4. was able to identify key points in the parent’s response and the question sequence was delivered fluently and with minimal delays. 5. incorporated clear, relevant probing / additional questions with confidence. | | | | | | | | | | | | | | | | | |
|  |  | 0 | | 1 | 2 | 3 | 4 | | 5 | | | 6 | 7 | 8 | | 9 | | 10 | |
|  |  | Omitted | |  |  |  |  | | Advanced beginner | | |  |  |  | |  | | Highly Proficient | |
| B | Empathy and active listening | A proficient practitioner:   1. demonstrated their ability to appropriately acknowledge the parent’s concerns throughout the interview. 2. when responding used congruent non-verbal communication effectively. 3. showed genuine interest in the parent’s response – non-verbal 4. was able to reflect and interpret the information the parent shared by including an accurate summary of the key points shared by the parent throughout the interview. | | | | | | | | | | | | | | | | | |
|  |  | 0 | 1 | | 2 | 3 | | 4 | | 5 | 6 | | 7 | | 8 | | 9 | | 10 |

Scored for each checklist item if it was covered at some point during the recording. I.e. if the order was different, I still scored each item. E.g. some participants would ask for triggers (H) before asking for a specific example of an incident (E). Or they would ask questions that relate to I throughout the recording.

| **Procedure skills** | | Description | | | | | | | | | | | | |
| --- | --- | --- | --- | --- | --- | --- | --- | --- | --- | --- | --- | --- | --- | --- |
| C | Began with open-ended question | A proficient practitioner:  (1) used a relevant, detailed question to start the conversation and to understand the parent’s concern  (2) included a reference to the child and their behaviour. e.g., “*What is concerning you about Ryan’s behaviour at the moment?”.* | | | | | | | | | | | | |
|  |  | 0 | 1 | 2 | 3 | 4 | 5 | | 6 | 7 | 8 | 9 | 10 | |
|  |  | Omitted |  |  |  |  | Advanced beginner | |  |  |  |  | Highly Proficient | |
| D | Helped the parent come up with a specific description of the problem behaviour | A proficient practitioner:   1. demonstrated an ability to clarify the parent’s concerns and identify specific details regarding the **onset** of the problem behaviour. 2. referred to the parent‘s description and used appropriate probing question(s) to specify the type of behaviour occurring. 3. clarified specific details of the child’s behaviour including verbal and physical responses and reactions to have a clear and full picture of the concern. 4. briefly summarized the description of the behaviour and confirmed their summary with the parent.   E.g., *“What do you mean by getting angry? What does he do exactly, what does it look like?” “When did these meltdowns start?”* | | | | | | | | | | | | |
|  |  | 0 | 1 | 2 | 3 | 4 | 5 | 6 | | 7 | 8 | 9 | | 10 |
| E | Asked the parent for a specific example of the problem behaviour | A proficient practitioner:   1. asked questions that generated details of the child’s behaviour 2. was able to direct the parent to a recent specific example. 3. provided appropriate probing questions, to clarify additional information regarding the example, i.e., duration, location, when, people present. 4. included questions to elicit the response of others (father, …) in the situation using relevant appropriate questions.   E.g., “*Can you describe a time this happened recently?”. “How did your husband react or did he say anything to Tom / Ryan?”* | | | | | | | | | | | | |
|  |  | 0 | 1 | 2 | 3 | 4 | 5 | 6 | | 7 | 8 | 9 | | 10 |
| F | Used more specific questioning techniques to identify the consequences of the behaviour | With reference to the example shared, a proficient practitioner skillfully included questions to:   1. elicit how the child’s behaviour ceased. 2. describe what caused the behaviour to stop including the consequences for the child and the parent. 3. appropriately identify the outcomes (learning) for the child and or parent.   E.g., “*What happened that helped him calm down or stop?”. “What do you think he is learning when this happens?”.* | | | | | | | | | | | | |
|  |  | 0 | 1 | 2 | 3 | 4 | 5 | 6 | | 7 | 8 | 9 | | 10 |
| G | Used more specific questioning to identify the antecedents of the behaviour | A proficient practitioner included detailed questions to specify what happened in the example event to capture relevant detail regarding:   1. what the parent said/did 2. the child’s response and 3. other’s involvement (father, …).   E.g. “*When you said no to the iPad, what did Tom do then?” “How did Jack respond when Tom said that?”.* | | | | | | | | | | | | |
|  |  | 0 | 1 | 2 | 3 | 4 | 5 | 6 | | 7 | 8 | 9 | | 10 |
| H | Used more specific questioning to obtain any additional useful information | Throughout the interview, a proficient practitioner:   1. used appropriate, well timed questions to ascertain the current parenting strategies being used by the parent. 2. asked to understand previous experience with parenting strategies and the father’s involvement. 3. asked additional questions to determine concerns the parent has regarding the stress associated with managing their child’s behaviour.   E.g., “What *have you tried to manage Tom’s behaviour? ” “How do you feel when dealing with Tom’s challenging behaviour?” “How have you and Bob discussed about ways to respond to Tom’s meltdowns?”* | | | | | | | | | | | | |
|  |  | 0 | 1 | 2 | 3 | 4 | 5 | 6 | | 7 | 8 | 9 | | 10 |
| I | Summarised the incident and checked the parent’s reaction. | To conclude the interview, a proficient practitioner:  (1) accurately summarised the example incident and relevant information shared by the parent.  (2) checked and confirmed the summary with the parent.  (3) adjusted their response when necessary, as the parent confirmed or corrected the key points.  E.g., “*I’ll summarise the information you’ve shared so that I can check I’ve understood”. “Is there anything I’ve missed or you would like to add?”.* | | | | | | | | | | | | |
|  |  | 0 | 1 | 2 | 3 | 4 | 5 | 6 | | 7 | 8 | 9 | | 10 |
| J | Checked for generality | A proficient practitioner checked for generality by:   1. asking if the behaviour described was typical or consistent across contexts. 2. clarified that the response shared by the parent in the example(s) was a typical parental response.   E.g. “Are the examples you described typical of what happens at home? Does It happen in other context as well (school, outside)?  *“Can I check, the way you described how you responded to the situation, is that how you would typically respond to this sort of behaviour at other times?” “Is this how you usually deal with Ryan / Tom’s similar behaviour? ”.* | | | | | | | | | | | | |
|  |  | 0 | 1 | 2 | 3 | 4 | 5 | 6 | | 7 | 8 | 9 | | 10 |
